# Activity of Ingavirin (6-[2-(1*H*-Imidazol-4-yl)ethylamino]-5-oxo-hexanoic Acid) Against Human Respiratory Viruses in *in Vivo* Experiments

**DOI:** 10.3390/ph4121518

**Published:** 2011-11-25

**Authors:** Vladimir V. Zarubaev, Angelica V. Garshinina, Nelly A. Kalinina, Anna A. Shtro, Svetlana V. Belyaevskaya, Alexander V. Slita, Vladimir E. Nebolsin, Oleg I. Kiselev

**Affiliations:** 1 Influenza Research Institute, 15/17 prof. Popova str., St. Petersburg 197376, Russia; 2 ValentaPharm, Ltd., 18 block 2, Gen. Dorokhova str., Moscow 119530, Russia

**Keywords:** influenza, parainfluenza virus, adenovirus, pandemic, Ingavirin, antiviral, animal model

## Abstract

Respiratory viral infections constitute the most frequent reason for medical consultations in the World. They can be associated with a wide range of clinical manifestations ranging from self-limited upper respiratory tract infections to more devastating conditions such as pneumonia. In particular, in serious cases influenza A leads to pneumonia, which is particularly fatal in patients with cardiopulmonary diseases, obesity, young children and the elderly. In the present study, we show a protective effect of the low-molecular weight compound Ingavirin (6-[2-(1*H*-imidazol-4-yl)ethylamino]-5-oxohexanoic acid) against influenza A (H1N1) virus, human parainfluenza virus and human adenovirus infections in animals. Mortality, weight loss, infectious titer of the virus in tissues and tissue morphology were monitored in the experimental groups of animals. The protective action of Ingavirin was observed as a reduction of infectious titer of the virus in the lung tissue, prolongation of the life of the infected animals, normalization of weight dynamics throughout the course of the disease, lowering of mortality of treated animals compared to a placebo control and normalization of tissue structure. In case of influenza virus infection, the protective activity of Ingavirin was similar to that of the reference compound Tamiflu. Based on the results obtained, Ingavirin should be considered as an important part of anti-viral prophylaxis and therapy.

## 1. Introduction

Respiratory viral infections constitute the most frequent reason for medical consultations in the World. They can be associated with a wide range of clinical manifestations ranging from self-limited upper respiratory tract infections to more devastating conditions, such as pneumonia. Therefore, the prevention and control of these infections remain major clinical goals. Currently, there are approximately 200 known respiratory viruses that can be grouped into one family of DNA viruses (*Adenoviridae*) and four families of RNA viruses (*Orthomyxoviridae*, *Paramyxoviridae*, *Picornaviridae* and *Coronaviridae*).

The influenza A virus (IAV) is a highly infective agent that causes acute pulmonary diseases. Outbreaks of highly pathogenic influenza virus infections and the appearance in 2009 of a new pandemic IAV have triggered renewed interest in influenza research. As of May 2010, more than 214 countries and overseas territories or communities have reported laboratory confirmed cases of IAV H1N1 2009, including more than 18,097 deaths [1]. Antiviral drugs occupy an important niche in the management of this disease [2,3]. They target virus-specific components and are an effective treatment when administered at the early stage of infection or soon after virus exposure [3].

Two main classes of anti-influenza drugs are currently accepted for chemotherapy of IAV. Derivatives of adamantane (amantadine and rimantadine) target the M2 ion channel of IAV and are not effective against influenza B virus [4]. Moreover, the rapid emergence of drug-resistance among influenza viruses since the mid-1990s have greatly compromised the effectiveness of these compounds [5]. All of the pandemic H1N1 viruses tested so far also appear drug-resistant [6].

Inhibitors of neuraminidase (NAIs, oseltamivir, zanamivir and peramivir) have a wider spectrum of activity that include both influenza A and B viruses [7]. Nevertheless, since 2007 rapid emergence and transmission of drug-resistant viruses have been observed [5,8,9]. Several strains resistant to NAIs were also isolated of pandemic H1N1 virus [10]. There is, therefore, a need both for identifying new and effective antivirals and for monitoring the susceptibility of circulating viruses to anti-viral compounds used in clinics.

Paramyxoviruses include important viruses associated with upper and lower respiratory tract infections in humans. Among them, human respiratory syncycial virus (RSV), and human parainfluenza viruses (HPIV) should be noted.

RSV is a major cause of lower respiratory tract disease in premature babies (≤35 months of gestation), infants less than 6 months old, and elderly institutionalized subjects. The outcome of RSV infection usually involves mild upper respiratory tract infections; however, more severe conditions, such as pneumonia and bronchiolitis occur in 25–40% of children. Approximately 1% of RSV-infected infants require hospitalization [11,12].

Four distinct serotypes of human parainfluenza viruses have been described [13]. These viruses can cause upper respiratory tract diseases in individuals of all age groups, although young children between 6 months and 3 years present more severe diseases [13].

Adenoviruses (AdV) are non-enveloped DNA-genome viruses. Despite multiorgan tropism, some types of AdV have a preferential tropism for the respiratory tract and can cause a wide range of respiratory symptoms, including coryza, pharyngitis, tonsillitis, bronchitis, and pneumonia. In general, AdV infections are mild or self-limited and resolve within two weeks without long-term complications. However, these viruses constitute an important cause of mortality and morbidity in immunocompromised patients including neonates and bone marrow transplant recipients [11].

Several compounds can be used for treatment of RSV, AdV and HPIV infection. Ribavirin inhibits viral replication by several mechanisms, including inhibition of viral polymerase, inhibition of 5′ cap formation of mRNA, and inhibition of IMP dehydrogenase leading to a decrease of intracellular GTP concentrations [14]. There are no approved therapeutic agents against AdV infections. However, some broad spectrum antivirals, like ribavirin, and nucleoside analog cidofoir have been used in the treatment of severe Ad infections in immunocompromised hosts [11]. In addition, fusion inhibitors, antisense oligonucleoides and steroids were used for this purpose, none of them have been approved for clinical application.

Previously, the low-molecular weight compound Ingavirin [6-[2-(1*H*-imidazol-4-yl)ethylamino]-5-oxohexanoic acid, also known previously as Ingamine] was shown to have anti-influenza activity against the influenza viruses A(H3N2), A(H5N1) and B in an animal model [15,16] and against the pandemic strains of influenza virus A/California/04/2009 and A/California/07/2009 [17–19]. In experiments with IAV H1N1 Ingavirin decreased the virus-induced cytopathogenic effect in cell culture 50 to 79% compared to control cells. Mice infected with either H3N2 or H1N1 (2009) IAVs and treated with Ingavirin demonstrated lower mortality (approx. 40%) and increased average lifespan (approx. 4 days) compared to placebo-treated animals. Taken together, these data suggest that Ingavirin is a prospective tool for the treatment of IAV infections, in particular those caused by the pandemic viruses. Moreover, Ingavirin demonstrated activity in *in vitro* and *in vivo* experiments against human adenovirus and parainfluenza virus [20,21]. Nevertheless, the exact mechanism of its clinical efficacy is far from complete understanding.

Here we summarize the results of studies into the protective activity of Ingavirin using the models of lethal influenza pneumonia caused by the pandemic influenza virus A(H1N1)2009, mild HPIV-caused pneumonia and disseminated adenovirus-induced infection and present a new data about its anti-viral activity regarding human respiratory viruses. Based on these results conclusions might be made about its range of activity and further application against specific diseases.

## 2. Materials and Methods

### 2.1. Compounds

**Figure 1:**
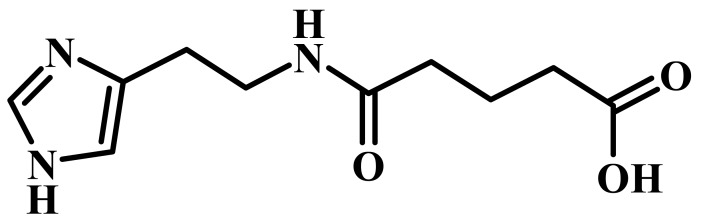
Structure of Ingavirin.

Ingavirin (6-[2-(1*H*-imidazol-4-yl)ethylamino]-5-oxohexanoic acid, Figure 1) was provided as a pure substance by the manufacturer (Valenta Pharmaceuticals, Moscow, Russia). Tamiflu (oseltamivir phosphate, LaRoche, Switzerland), 6-azacytidine (Institute of Molecular Biology and Genetics, Kiev, Ukraine) and ribavirin (ICN Biochemicals, USA) were used in the experiments as reference drugs.

### 2.2. Viruses

Influenza virus A/California/07/09 (H1N1) was obtained from the collection of viruses from the Influenza Research Institute. Prior to the experiments, the virus was adapted to mice by three serial passages in the lung tissue of mice, followed by a subsequent passage through the allantoic cavity of 10–12 day old chicken embryos and a final passage through mice [22]. Lung homogenate in nine volumes of sterile phosphate-buffered saline was used as an infecting material in further experiments.

Human parainfluenza virus (hPIV) type 3 (strain HA1) and human adenovirus (AdV) type 5 were obtained from the collection of viruses from the Influenza Research Institute and propagated in MA-104 (ATCC CRL-2378) or HEp-2 (ATCC CCL-23) cells, correspondingly, at 36 °C in 5% CO_2_.

### 2.3. Animals

Female Balb/c mice, 16–20 grams, from the Rappolovo laboratory animal breeding farm were used in the experiments with influenza virus. Syrian hamsters bred in Influenza Research Institute were used for experiments with hPIV and AdV. The animal experiments were planned in accordance with the principles of laboratory animals care (*Guide for the Care and Use of Laboratory Animals*, National Academy Press: Washington, DC, USA, 1996) and approved by the Institutional Ethical Committee.

### 2.4. Virus Titration

Prior to the studies of the protective activity of Ingavirin in animals, mouse-adapted influenza virus was titrated for its lethal effect. For this purpose mice (10 in each experimental group) were inoculated intranasally under anesthesia with 50 μL of serial decimal dilutions (10^−1^ to 10^−5^) of the lung homogenate of virus-infected mice. The dilution that caused death of 50% of the animals in 14 days post infection (LD_50_) was calculated as described previously [23] and used for subsequent experiments.

### 2.5. Protective Activity of INGAVIRIN

For evaluation of the anti-influenza activity of Ingavirin *in vivo*, mice were infected with five LD_50_ (20 mice) or one LD_50_ (30 mice) of the previously titrated virus (see “Virus titration” section). Ingavirin was diluted in saline to the doses of 15, 20 or 30 mg/kg body weight/day and applied orally via gavage once a day on day one, two, three, four and five post infection (p.i.). To study the effect of treatment schedule on the protective activity, separate groups of animals were treated twice (days 1 and 2 p.i.) with 30 mg/kg Ingavirin following by three doses of 15 mg/kg (days 3, 4 and 5 p.i.). The total amount of Ingavirin received by animals in this group was equal to that in the group of 20 mg/kg. The reference drug Tamiflu (final dose 20 mg/kg body weight) was dissolved in saline and applied to 20 mice (five LD_50_) or 30 mice (one LD_50_) orally in a volume of 200 μL. Control animals were treated with sterile saline.

Animals in all experimental groups were weighed daily. The mortality in each group of animals was calculated. Each group was checked daily for dead animals for two weeks post inoculation. Based on the data received, percent of mortality, index of protection (ratio of mortality in the control group over mortality in the experimental group) and mean day of death (MDD) were calculated.

On day three p.i., ten mice from each group infected with one LD_50_ of the virus were sacrificed, their chest opened and lungs isolated. Five lungs were used for virus titration and five others for histological examination (see “Histological examination” section).

In order to determine infectious titer of the virus in lung tissue, lungs were homogenized in ten volumes of sterile phosphate-buffered saline. Serial dilutions (10^−1^–10^−7^) were prepared from each homogenate. MDCK cells grown in 96-well plates were inoculated with 0.2 mL of each dilution and incubated at 36 °C for 48 hours in 5% CO_2_. After incubation, supernatant was harvested and tested for the presence of influenza virus by mixing the fluid in round- bottom wells with equal volumes of a 1% suspension of chicken erythrocytes in saline. Virus titer in the lungs was considered the final dilution when it caused a positive hemagglutination reaction in the well, and the virus titer is expressed in log_10_EID_50_/20 mg tissue. The activity of the compounds was evaluated by their ability to decrease the infectious titer of the virus in lung tissue.

For the study of anti-hPIV activity of Ingavirin, four to five weeks old Syrian hamsters were infected with 0.05 mL (10^4^ TCID_50_) of human parainfluenza virus (hPIV) intranasally as described in [24]. Ingavirin was used as described above. On day 3 and 7 p.i. animals were sacrificed, and their lungs used for virus titration in MA-104 cells and histological analysis (see below), respectively. Virus titer was determined by ELISA using anti-hPIV monoclonal antibodies (PPDP Ltd., St. Petersburg, Russia).

Anti-AdV activity of the compound was tested as described previously [25].

### 2.6. Histological Examination

Lungs of animals were fixed in 4% PBS-buffered formaldehyde, dehydrated in graded ethanol and embedded in paraffin. Four-micrometer sections were cut and stained with haemotoxylin-eosin.

In case of influenza infection, cells of bronchial epithelium were divided into four morphologically distinct categories: (i) intact cells without signs of virus replication; (ii) cells with initial stages of formation of virus-specific inclusions; (iii) cells with advanced virus inclusions; and (iv) dead cells which looked like gaps between other cells with basal membrane denudation. The rates of each category of cells among cells of bronchial epithelial layer were calculated. The morphometric values were evaluated by two independent observers.

## 3. Results

### 3.1. Influenza Infection

Inoculation of animals with an adapted influenza virus led to development of influenza pneumonia. The clinical signs of the disease were typical for severe influenza infection and included ataxia, tremor, short breath, as well as decrease of water and food consumption leading to weight loss. On day 15 p.i. death of 55–90% of infected animals was observed, depending on infecting dose of the virus.

**Figure 2:**
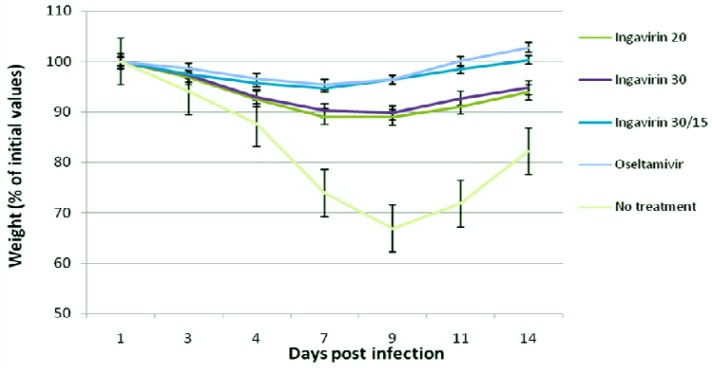
Dynamics of body weight of mice in the course of pneumonia caused by influenza virus A/California/7/09 (H1N1)v.

The protective activity of Ingavirin was evaluated when applied once a day for five days after virus inoculation. No non-specific mortality was observed in control groups of non-infected animals treated with saline and non-infected mice treated with Ingavirin. Ingavirin application resulted in decreased mortality (to 18–67%) as well as an increase in the mean day of death (1.2–4.1 days) compared to the control animals (depending on the dose of virus and compound). Mice treated with the reference compound Tamiflu also demonstrated significantly lower mortality (index of protection 80%) and prolongation of mean lifespan (up to 5.4 days) compared to control values (Table 1). In addition, treatment of the animals with Ingavirin, similarly to the reference compound Tamiflu, resulted in normalization of weight dynamics of the animals (Figure 2), leading to similar weight profiles compared to non-infected animals.

As shown by virus titration from lungs of mice, on day three p.i. the virus replicated in the lung tissue up to 10^5.1^ EID_50_/20 mg tissue. Application of the reference compound Tamiflu decreased the viral titer approximately 320-fold (10^2.6^ EID_50_/20 mg tissue). Treatment of the animals with Ingavirin also resulted in a decrease of the virus' titers (approximately 10^3.5^ EID_50_/20 mg), which is statistically identical to the activity of oseltamivir (Table 1).

**Figure 3:**
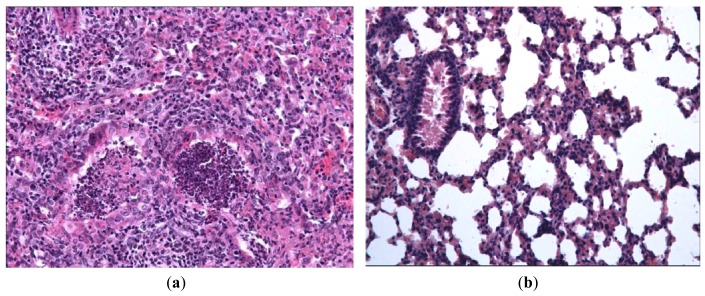

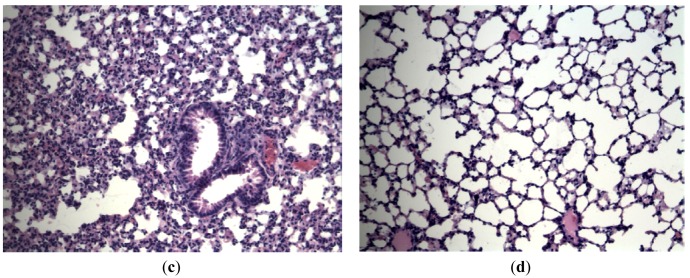
Pathologies of mice intranasally inoculated with mouse-adapted influenza virus A/California/7/09 (H1N1)v on day 3 p.i. (**a**) Lungs of a placebo-treated mouse. Large area of pneumonia with intense exudates in bronchial lumen, severe neutrophilic-to-histiocytic alveolitis; (**b**) Mild lymphocytic peribronchitis with mild alveolitis in a mouse treated with 30 mg/kg body weight of ingavirin. Cells of bronchial epithelium are intact, mild exudates in bronchial lumen; (**c**) Mild lymphocytic peribronchitis with alveolitis in a mouse treated with 20 mg/kg oseltamivir; (**d**) Lungs of intact mice. No signs of inflammation or cell destruction. Hematoxylin-eosin, ×200.

In order to evaluate the effect of Ingavirin on the structure of the lung tissue, morphology analysis was performed on day three p.i. Lungs of virus-infected mice were consolidated and edematous. All infected mice had exudative diffuse alveolar damage with interstitial edema, fibrinous exudates in their alveoli, inflammatory infiltration, bronchiolar epithelial necrosis and desquamation. Cells of bronchial and bronchiolar epithelium contained viral inclusions or were absent with denudation of the basal membrane [Figure 3(a)].

Application of Ingavirin, similar to the reference compound Tamiflu, resulted in normalization of lung tissue structure, in particular, restriction of edema and alveolar damage, decrease in the amount of debris in the bronchial lumen and protection of bronchial epithelium from death [Figure 3(b), Table 1].

To study the mechanism of protective activity of Ingavirin, we looked more closely to the cells of bronchial epithelium that are considered a primary targets for influenza virus. Morphometric analysis of cells revealed that almost 100% of epithelial cells of intact mice were represented by intact cells.

**Figure 4:**
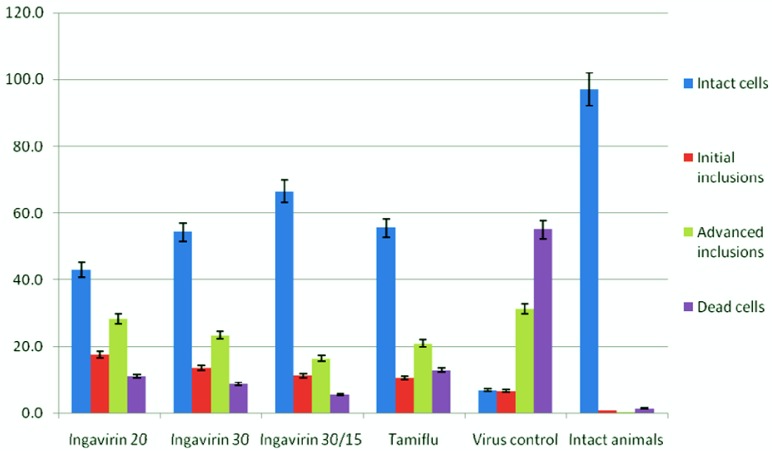
Cytoprotective activity of Ingavirin in cells of bronchial epithelium after infecting with mouse-adapted influenza virus A/California/7/09 (H1N1)v (day 3 p.i.).

Inoculation with influenza virus led to increasing of the rate of cells of three other groups (*i.e.*, cells with initial inclusions, advanced inclusions and dead cells), suggesting a strong cytotoxic action of the virus. Application of both Ingavirin and reference compound oseltamivir strongly decreased the number of dead cells and increased the rate of intact cells of bronchial epithelium. From these results we suggest that this compound is able to protect cells against virus- induced damage (Figure 4).

### 3.2. Parainfluenza Virus Infection

Infecting of Syrian hamsters with human parainfluenza virus (hPIV) results in non- fatal mild bronchitis, bronchiolitis and pneumonia. The virus can be recovered from the lungs on day 3 p.i., and specific tissue damage can be observed on day 7.

As shown by virus titration, application of both ingavirin and reference compound ribavirin led to reduction of infectious titer of hPIV in lung tissue. Ribavirin demonstrated the highest activity in decreasing the titer although both doses of Ingavirin also led to statistically significant reduction (Table 2).

**Figure 5:**
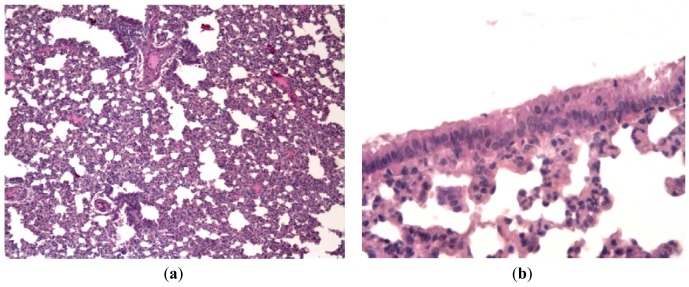

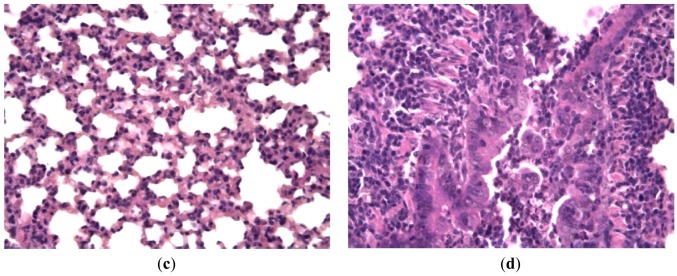
Structure of lung tissue of Syrian hamsters on day 7 after inoculation with hPIV-3 with (**c**,**d**) or without (**a**,**b**) treatment with Ingavirin. **a**, **c**—Alveoli, **b**, **d**—Bronchial epithelium. Hematoxylin-eosin, ×100 (**a**), ×400 (**b**,**c**,**d**).

Hamsters infected with hPIV showed no observable evidence of illness prior to sacrifice, but histologically there was consistent production of pneumonia. Lung lesions consisted of scattered endobronchial exudates composed of mononuclear and polymorphonuclear cells, peribronchial and perivascular round cell infiltrates, and wide areas of interstitial pneumonia. Bronchial epithelium had a specific appearance containing groups of tall giant cells protruding into the bronchial lumen [Figures 5(a,b)]. Animals treated with Ingavirin and ribavirin demonstrated almost normal lung tissue architecture with few infiltrating cells [Figures 5(c,d)].

### 3.3. Human Adenovirus Infection

As human adenoviruses do not cause airborne respiratory infection in animals, we studied the anti-viral activity of ingavirin using previously developed model of disseminated adenoviral infection of newborn Syrian hamsters caused by human adenovirus type 5 [25]. Similar to hPIV infection, protective activity was evaluated by virus titration in target organs and histological analysis of tissue architecture.

Subcutaneous infecting of newborn Syrian hamsters resulted in the replication of the virus in lungs and livers of animals. The results of virus titration in HEp-2 cells on day three p.i. are summarized in Table 3.

As can be seen, treatment of AdV infection with reference compound 6-AC resulted in restriction of virus replication both in liver and lungs of animals that is in agreement with our previous results [25]. Application of ingavirin led to moderate (approx. one decimal order), but statistically significant reduction of virus titer both in liver and lungs of animals.

**Figure 6:**
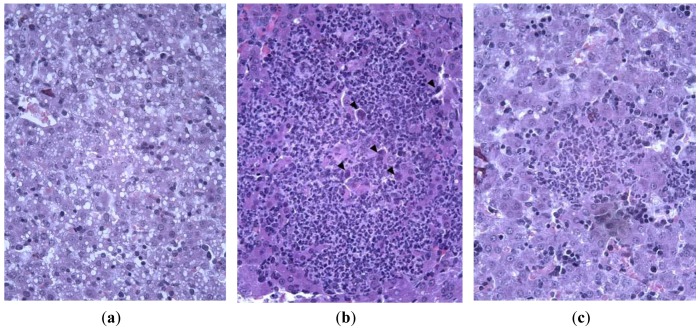
Pathology of AdV-induced hepatitis in the liver of newborn Syrian hamsters on day 3 p.i. Large focus of inflammation with numerous AdV-infected cells (arrowheads), vacuolization of hepatocytes (**a**,**b**) in control animals, small focus of inflammation and intact parenchyma (**c**) in Ingavirin-treated animals. Hematoxylin-eosin, ×400.

Histological investigation of liver of infected animals revealed *foci* of necrosis. Morphologically, they were the areas of destruction of the parenchyma caused by specific lesion of hepatocytes and non-specific destruction of tissue due to a local inflammatory reaction. Specific lesions of liver cells were manifested in the augmentation of cell nuclei, their deformation and appearance of eosinophylic and basophylic virus-specific nuclear inclusion bodies. The reactive alterations of tissue were due to inflammatory destruction of hepatocytes and tissue infiltration with leukocytes. Infected animals treated with ingavirin had smaller *foci* of inflammation and intact-looking hepatocytes, in contrast to highly vacuolized hepatocytes in control animals (Figure 6).

In order to quantify the protective effect a Ingavirin, we measured the size of the foci of necrosis in liver and counted the number of AdV-infected cells within each focus (Table 4).

As shown by morphometry analysis, application of Ingavirin decreased mean size of *foci* of virus-induced inflammation and strongly reduced the number of infected cells. Interestingly, the effect of higher dose of the drug (45 mg/kg/day) was less than that of lower dose (30 mg/kg/day) suggesting a specific mode of this compound's activity.

## 4. Discussion

In the present study, we showed a protective effect of the low-molecular weight compound Ingavirin against lethal influenza virus infection caused by the pandemic influenza virus A (H1N1) in mice and non-fatal pathologies of Syrian hamsters caused by human parainfluenza virus and human adenovirus. Effects of the dose on the protective activity of the compound and virus replication in tissue were investigated. The protective action of Ingavirin was shown as a reduction of infectious titer of the virus in the lung tissue, prolongation of life of infected animals, normalization of weight dynamics in the course of disease, lowering of mortality of treated animals compared to a placebo control and normalization of lung and liver tissue structure. In case of influenza infection, the protective activity of Ingavirin appeared similar to that of the reference compound Tamiflu.

In our experiments, Ingavirin demonstrated the protective activity against lethal influenza pneumonia in mice. At some doses, the protective effect was equal to the activity of oseltamivir, which is an internationally accepted drug proved to be effective against this IAV. In general, our results are in good agreement with previously obtained data [15–19] where Ingavirin was shown to have a protective effect against the influenza viruses A and B.

Krug and Aramini [26] suggested that two possible domains of the influenza virus nucleoprotein (NP), one located in a tail loop and another in an RNA-binding groove found between the head and body domains at the exterior surface of the NP trimer, represent the potential antiviral targets. The tail loop-located domain is crucial for oligomerization of NP that is, in turn, necessary for efficient transcription and replication of viral genome. Inactivation of this domain, therefore, might be effective for suppressing the virus replication. Indeed, some experiments suggest Ingavirin targets the influenza nucleoprotein (NP). In a recent study [27] ingavirin was shown to interact with the influenza virus NP, thus preventing the NP oligomerization necessary for viral replication. In third study, several analogues of mycalamide A were identified as NP-directed inhibitors of influenza virus replication [28]. These compounds were shown to bind to the N-terminal 13-amino acid tail, which mediates the nuclear transport of NP and its binding to viral RNA. Moreover, Kao *et al.* [29] reported the identification of a small-molecule compound, nucleozin, that triggers the aggregation of NP and inhibits its nuclear accumulation. Nucleozin impeded influenza A virus replication *in vitro* with a nanomolar concentration and protected mice challenged with lethal doses of avian influenza A H5N1. These data suggest that compound binding to this target may inhibit viral replication by inhibiting the functions of the NP. Neither mycalamide A analogues nor nucleozin demonstrate no structural similarity with Ingavirin, leading us to hypothesize that other domain(s) of the viral NP may be involved in the interaction with Ingavirin.

From another hand, application of Ingavirin was shown to result in change of the morphology of virions detected in bronchoalveolar lavage of infected mice [30]. Control animals produced mostly spherical virions while in Ingavirin-treated animals presumably filamentous particles of reduced infectivity were formed. These results suggest that ingavirin might interfere with the process of virus assembly and/or budding leading to reduction of viral load.

In our study the activity of oseltamivir appeared lower than in similar experiments of Smee *et al.* [31]. This might be connected with a higher virus dose, different virus used in our experiments and different schedule of application of the drug (once a day instead twice a day in [31]).At the same time, direct anti-viral effects of Ingavirin detected by a decrease of the virus' infectious titer in lung tissue appeared ten times less than that of Tamiflu despite similar level of protection from lethality (Table 1). This contradiction could indicate that other mechanisms, in addition to a direct anti-viral activity, might contribute to the resulting protection of animals from lethality caused by the IAV.

Influenza virus infection ranges in severity from an asymptomatic infection to a serious illness with systemic features. Severe influenza is manifested by virus-specific reactions with further development of reactive processes. These processes are induced by the replicating virus in the target cells and realized through host mechanisms, including immune reactions, oxidative stress and other free radical processes, enhanced proteolytic activity, sharp elevation of the level of proinflammatory cytokines, and more [32].

In clinics, one of the main reasons for severe and complicated influenza pneumonia, including fatal cases, is late and/or inadequate treatment [33,34]. In these cases, the course of the disease is driven by mechanisms that are initially induced by the virus but ultimately realized by the host, including, in particular, severe inflammation (“cytokine storm”) [35–37]. Experiments using influenza virus-infected knockout mice with inactivated genes in the inflammatory pathways, such as interleukin 1α/β, macrophage chemokine receptors CCR5 and CCR2, cyclooxygenase 1 and 2 [38–40] have clearly demonstrated that in addition to the level of virus' replication in the lungs, the intensity of the host reactions contribute significantly to the course and outcome of the disease. In severe cases of influenza, therefore, both direct anti- viral and pathogenetic drugs should be included into complex therapy, in particular those compounds that restrict the cytokine storm, lung edemas, inflammation and tissue damage [41]. For example, recently the high protective activity of 7-hydroxycoumarin (7-HC) was demonstrated [42]. 7-HC was demonstrated to possess anti-viral properties owing to its ability to decrease the level of proinflammatory cytokines in infected animals, thus alleviating the severe influenza infection. At the same time 7-HC did not reduce the level of virus replication in plaque reduction assays suggesting that its protective activity, including decrease of virus replication in mouse lungs, is of a complex nature and may be mediated by cell signaling and reactive pathways. One could suggest that, in addition to the ability to directly decrease the level of virus replication in the lungs (Table 1), Ingavirin might have similar properties based on the results of the mouse lung morphology examinations showing that Ingavirin treatment significantly reduced the degree of tissue damage, inflammation and edema (Figure 3, Table 1). Further studies into the effects of Ingavirin on different pathogenetic pathways would be useful for understanding its mechanism of activity. Ingavirin should be considered as an important part of anti-influenza prophylaxis and therapy, in particular in severe cases of the disease.

In our experiments Ingavirin also demonstrated anti-viral activity against two other viruses used, hPIV and AdV [19,20]. As these viruses are phylogenetically distinct from each other and there are no viral components common to all three viruses used in the study, it may be concluded that this drug targets the components and pathways responsible for development of cell and tissue pathology during viral infection. Indeed, in all three cases it demonstrated high degree of cytoprotection and ability to normalize the architecture of the tissue. Its application prevented the death of influenza virus-infected cells of bronchial epithelium, hPIV-induced cytopathology in lungs and virus-induced vacuolization of hepatocytes during AdV infection in hamsters. Moreover, in our previous experiments [21] we demonstrated the ability of Ingavirin to prevent AdV-induced cell damage in culture. Despite formation of typical intranuclear virus-specific inclusions, Ingavirin-treated cells did not develop vacuoles in cytoplasm and other morphological signs of cytopathogenicity. Therefore, in addition to other mechanism(s) of anti-viral action, Ingavirin possesses a cytoprotective activity that prevents destruction of infected cells and maintains the function of the target organ thus minimizing the virus-induced tissue damage and toxicity symptoms in the course of disease.

It should be noted that no toxicity of Ingavirin was observed at the doses up to the doses of 3,000 mg/kg. Also, no signs of embryotoxicity was observed in previous studies [43]. For comparison, LD_50_ for Tamiflu was estimated as 100–250 mg/kg depending on the route and schedule of inoculation and species of animals [44]. Moreover, in clinical trials Ingavirin did not demonstrate any side effects when applied to influenza-infected patients [45]. Ingavirin, therefore, can be considered as non-toxic compound with low risk of overdose.

## 5. Conclusions

Taken together, our data suggest that Ingavirin is a non-toxic broad spectrum antiviral with complex mechanism of action. Further study of fine mechanism of its protective activity would allow to optimize the drug structure and probably develop new class of compounds for prophylaxis and treatment of viral infections.

## Figures and Tables

**Table 1:** Protective activity of Ingavirin against influenza A (H1N1) 2009. p < 0.05 values are indicated in bold.

**Preparatio**	**Virus dose, LD_50_**	**Survive d/total infected**	**Mortality, %**	Medium day of death * (comparing to control)	**Index of protection, %**	**Virus titer in the lungs (log_10_EID_50_/20 mg tissue) on day 3 p.i.**	**Degree of lung edema and infiltration**
Ingavirin	1	11/20	45	10.9 ± 0.7(+1.2)	18	3.8 ± 0.3 *	**1.5 ± 0.1** *
20 mg/kg	5	10/20	50	**9.1 ± 0.9 (+3.4)**	44	N/D **	N/D
Ingavirin	1	16/20	20	**12.9 ± 0.8(+3.2)**	64	3.5 ± 0.3	**1.5 ± 0.2**
30 mg/kg	5	14/20	30	**9.8 ± 0.5 (+4.1)**	67	N/D	N/D
Ingavirin	1	16/20	20	**13.4 ± 0.7 (+3.7)**	64	3.4 ± 0.3	**1.4 ± 0.1**
30/15 mg/kg	5	14/20	30	**12.0 ± 0.5 (+6.3)**	67	N/D	N/D
Oseltamivr	1	18/20	10.0	**13.1 ± 0.8(+3.4)**	81	2.6 ± 0.3	**1.7 ± 0.2**
20 mg/kg	5	15/20	25.0	**11.1 ± 1.0(+5.4)**	72	N/D	N/D
Control	1	9/20	55.0	9.7 ± 1.0	---	5.1 ± 0.2	2.7 ± 0.1
(no drugs)	5	2/20	90.0	5.7 ± 0.6	---	N/D	N/D

* mean ± SEM;

** Not determined.

**Table 2:** Infectious activity of hPIV in lung tissue of Syrian hamsters after application of Ingavirin.

**Drug**	**Virus titer (log_10_EID_50_/20 mg tissue)**	**p**
Ingavirin 30 mg/kg	3.2 ± 0.2	0.018
Ingavirin 30/15 mg/kg	2.7 ± 0.3	0.016
Ribavirin	2.3 ± 0.3	0.001
Control (no drugs)	3.8 ± 0.2	1.000

**Table 3:** Infectious activity of AdV in liver and lungs of Syrian hamsters after application of ingavirin.

**Drug, dose (mg/kg/day)**	**Virus titer in liver (log_10_EID_50_/20 mg tissue)**	**Virus titer in lungs (log_10_EID_50_/20 mg tissue)**
Ingavirin 15	3.8 ± 0.1 (p = 0.099)	4.1 ± 0.3 (p = 0.319)
Ingavirin 30	3.4 ± 0.2 (p = 0.022)	3.5 ± 0.2 (p = 0.016)
Ingavirin 45	3.7 ± 0.2 (p = 0.110)	3.7 ± 0.3 (p = 0.088)
6-azacytidine	2.7 ± 0.3 (p = 0.003)	2.9 ± 0.4 (p = 0.000)
Control (no drugs)	4.3 ± 0.3	4.5 ± 0.2

**Table 4:** Effect of Ingavirin on the course of AdV-induced hepatitis in newborn Syrian hamsters.

**Drug, dose (mg/kg/day)**	**Volume of focus, mm^3^ × 10^−5^**		**Number of infected cells within one focus**	
	**Mean ± SE**	**p**	**Mean ± SE**	**p**
Ingavirin 15	53.2 ± 5.3	0.000	2.9 ± 0.9	0.000
Ingavirin 30	50.8 ± 4.4	0.000	1.9 ± 1.5	0.000
Ingavirin 45	54.3 ± 5.0	0.000	2.8 ± 2.2	0.000
Control (no drugs)	84.4 ± 6.9	1.000	7.1 ± 0.3	1.000
